# Schwann Cell Migration Induced by Earthworm Extract via Activation of PAs and MMP2/9 Mediated through ERK1/2 and p38

**DOI:** 10.1093/ecam/nep131

**Published:** 2011-01-04

**Authors:** Yung-Ming Chang, Ying-Ting Shih, Yueh-Sheng Chen, Chien-Liang Liu, Wen-Kuei Fang, Chang-Hai Tsai, Fuu-Jen Tsai, Wei-Wen Kuo, Tung-Yuan Lai, Chih-Yang Huang

**Affiliations:** ^1^Graduate Institute of Chinese Medical Science, China Medical University, Taiwan; ^2^School of Chinese Medicine, China Medical University, Taiwan; ^3^Emergency Department, China Medical University Hospital, Taiwan; ^4^Department of Neurosurgery, Chia-Yi Christian Hospital, Chia-Yi, Taiwan; ^5^Department of Healthcare Administration, Asia University, Taiwan; ^6^Department of Pediatrics, Medical Research and Medical Genetics, China Medical University, Taiwan; ^7^Department of Biological Science and Technology, China Medical University, Taiwan; ^8^School of Post-Baccalaureate Chinese Medicine, China Medical University, Taiwan; ^9^Graduate Institute of Basic Medical Science, China Medical University, Taichung 404, Taiwan; ^10^Department of Health and Nutrition Biotechnology, Asia University, Taichung, Taiwan

## Abstract

The earthworm, which has stasis removal and wound-healing functions, is a widely used Chinese herbal medicine in China. Schwann cell migration is critical for the regeneration of injured nerves. Schwann cells provide an essentially supportive activity for neuron regeneration. However, the molecular migration mechanisms induced by earthworms in Schwann cells remain unclear. Here, we investigate the roles of MAPK (ERK1/2, JNK and p38) pathways for earthworm-induced matrix-degrading proteolytic enzyme (PAs and MMP2/9) production in Schwann cells. Moreover, earthworm induced phosphorylation of ERK1/2 and p38, but not JNK, activate the downstream signaling expression of PAs and MMPs in a time-dependent manner. Earthworm-stimulated ERK1/2 and p38 phosphorylation was attenuated by pretreatment with U0126 and SB203580, resulting in migration and uPA-related signal pathway inhibition. The results were confirmed using small interfering ERK1/2 and p38 RNA. These results demonstrated that earthworms can stimulate Schwann cell migration and up-regulate PAs and MMP2/9 expression mediated through the MAPK pathways, ERK1/2 and p38. Taken together, our data suggests the MAPKs (ERK1/2, p38)-, PAs (uPA, tPA)-, MMP (MMP2, MMP9) signaling pathway of Schwann cells regulated by earthworms might play a major role in Schwann cell migration and nerve regeneration.

## 1. Introduction

Nerve regeneration is a complex physiological response that takes place after injury. Neurons can be separated into central and peripheral nervous systems, which have different anatomical structures and regeneration ability. In mammals, the central neurons without a myelin sheath are very difficult to regenerate. In contrast, the peripheral nervous system (PNS) with a myelin sheath exhibits easier regrowth [1]. The regrowth ability results from the intrinsic neuronal activities and surrounding non-neuronal properties in which Schwann cells provide an essentially supportive activity for neuron regeneration. Schwann cells differentiate into the myelin sheath of the PNS and can proliferate and migrate into the distal end in the injured nerve area to support axonal regrowth [2]. It has also been reported that Schwann cell migration, which also occurs at the proximal end of the injury area, provides a guide for regenerating axons by interacting with nerve fibers or basal lamina [3]. Schwann cell migration is crucial for successful axonal elongation [3, 4]. Moreover, peripheral nerve injury locally activates Schwann cells and macrophages to synthesize a cocktail of neurotrophic factors, adhesion molecules, cytokines and growth-promoting surface molecules [5, 6]. However, the factors that regulate Schwann cell migration and their signaling mechanisms remain unclear.

The mitogen-activated protein kinase (MAPK) family is a crucial regulator of the pathways involved in cell proliferation [7] and migration [8]. Extracellular signal-regulated protein kinase (ERK) is the most extensively studied MAPK [9]. Recently, several studies found that after nerve injury, which increased ERK [10] phosphorylation, requires ERK to promote neurite outgrowth [11]. However, ERK has also been implicated in the migration of various cell types, including fibroblasts and carcinoma cells [12, 13], but not in Schwann cells. Interestingly, to promote migration, growth cones secrete proteases that are thought to dissolve cell-cell and cell-matrix adhesions during peripheral nerve regeneration. These proteases include the plasminogen activators (PAs), tissue PA (tPA) and urokinase PA (uPA) and their substrate, plasminogen [14]. Plasmin, activated by tPA or uPA, can activate MMP-9 and MMP-2 [15]. Many experiments have determined that after injury PA expression is rapidly induced in neurons [14, 16]. A lack of plasminogen activators affects MMP-9 and MMP-2 activity [17]. However, little is known about Schwann cell migration using MEK/ERK signaling pathways to active PAs and MMPs. In addition, c-Jun NH2-terminal kinase (JNK) and p38 are other members of the MAPK super family. Accumulating evidence has also implicated the JNK and p38 pathway in cell migration regulation [18]. The expression of matrix-degrading proteolytic enzymes (PAs and MMPs) could be regulated by JNK [19] and p38 [20] signal transduction pathways to promote migration in cells. Collectively, we investigated the important role of MAPKs in the regulation of PAs and MMPs activity in response to earthworm stimuli. Therefore, enhancing the migration ability of Schwann cells might be another potential approach to neuron regeneration.

With a history of several thousand years, the pharmacology and clinical application of traditional Chinese medicine has been well documented. Recently, biomedical material science combined with Chinese herbal medicine has been applied to nerve regeneration studies. For example, Schwann cells filled into a silicon rubber chamber can bridge a 15-mm gap in rat sciatic nerves. Several Chinese medicines have been identified as enhancing neuron regeneration [21]. Therefore, neuron regrowth induction using Schwann cells and herbal medicine has good potential for treating injured nerves. The earthworm is a widely used Chinese herbal medicine [22]. It has dense nutritional content because of their soil-based origin [23]. Extracting medicinal compounds from the earthworm has traditionally been practiced by indigenous people throughout the world, more particularly in Asia [24]. Previous earthworm studies have shown its antimicrobial [25], hepatoprotective [23], anticancer [26] and scar wound-healing characteristics [27]. The anti-inflammatory activity together with antioxidant properties seems to be due to the high polyphenolic content in earthworm tissue [28]. Moreover, crude earthworm extract has a thrombolytic effect that could significantly promote blood circulation to remove stasis [29]. Lumbrokinase extracted from the earthworm has been used to treat stroke and cardiovascular diseases [30]. Lumbrokinase is a group of proteolytic enzymes [31] that include a plasminogen activator and plasmin [32]. It can dissolve fibrin directly and also activate plasminogen [33]. In healthy human volunteers, oral-administered earthworm powder increased levels of tPA and fibrinolytic activity [34]. Earthworm tissue homogenates have revealed a glycolipoprotein mixture referred to as G-90, composed of macromolecules. G-90 possesses several growth factors and also participates in tissue regeneration and wound healing [35]. *In vivo* experiments have also found that a mixed prescription of liquid extracted from earthworm more obviously improves peripheral nerve regeneration than icariin [36]. Verified earthworm nerve regenerating and movement enhancing effects on Schwann cells are unknown. There is as yet no conclusive explanation for the possible molecular mechanism involved in Schwann cell migrating effects. Therefore, this study investigates the molecular mechanism and signaling pathways of earthworm extract in neuron regeneration. Our results indicate that earthworm extract promotes Schwann cell migration through the activation of PAs and MMPs, namely ERK1/2 and p38MAPK pathways.

## 2. Methods

### 2.1. Earthworm Extraction

Spray-dried earthworm powder, *Pheretima aspergillum (Annelida, Oligochaeta, Lumbricidae)*, was purchased from Wann-Guo Pharmaceutical Co., Ltd., Tainan, Taiwan, R.O.C. Two grams of earthworm powder was dissolved in 10 ml of 70% ethanol and left at room temperature for 24 h. The next day the clear supernatant fraction was collected after centrifugation at 2000 rpm for 20 min. The solvents were then removed using a water bath at 37°C for 4 h. The extract was then centrifuged for 5 min at 5000 rpm at 4°C. The supernatant was then filtered through a 0.22 *μ*m microspin filter just prior to the experiments. The concentrations used in the *in vitro* model were 0, 31.25, 62.5, 125, 250, 500 and 1000 *μ*g/ml for RSC96 cell treatment. All solutions were stored at –80°C.

### 2.2. Cell Culture and Treatments

RSC96 cells were purchased from American Type Culture Collection (ATCC) and cultured in Dulbecco's modified Eagle's medium (DMEM) supplemented with 10% fetal bovine serum (FBS), 4 mM _L_-glutamate, 1.5 g/l sodium bicarbonate and 1% non-essential amino acids (NEAAs) in a humidified atmosphere of 5% CO_2_ and 95% air. RSC96 cells cultures were treated at the indicated times or concentrations with earthworm extract.

### 2.3. Inhibitor

RSC96 cells were treated with several inhibitors, including U0126 (MEK1 and MEK2 inhibitor; Promega) and SB203580 (p38 MAP kinase inhibitor; Promega).

### 2.4. MTT

Cell viability was estimated using a colorimetric assay based on tetrazolium dye (MTT) conversion into a blue formazan product. All procedures were described in our previous study [37]. After harvesting and washing twice with PBS, the cells were cultured in phenol red-free DMEM (1 ml) with MTT (0.5 mg/ml) at 37°C for 4 h. The cells were then incubated in iso-propenol (1 ml) with shaking for 10 min, aspirated and measured spectrophotometerically at 570 nm.

### 2.5. Migration

We used a Boyden chamber and polyvinyl-pyrrolidone-free polycarbonate membranes with 8-*μ*m pores (Neuro Probes, Inc.) for the migration assay. The bottom wells of the chamber were filled with 10% FBS DMEM medium. The wells were covered with a membrane sheet, in which serum-free medium was added into the top chamber. Membranes were then stained with Giemsa stain (Sigma). Cells that migrated through the membrane were counted using a counting grid fitted into a phase contrast microscope eyepiece.

### 2.6. Wound Healing

Cells were initially seeded uniformly in 60-mm culture plates with an artificial “wound” carefully created at 0 h, using a sterile P-200 pipette tip to scratch the subconfluent cell monolayer to make an approximately 1.0-mm gap. After 24 h of culture with 125 *μ*g/ml earthworm concentration, the cell migration was calculated by counting cell numbers that had advanced into the cell-free space randomly selected from an area at the initial wound border. Photographs were taken of the wounded regions using an inverted Olympus microscope.

### 2.7. Western Blot

Cultured RSC96 cells were scraped and washed once with PBS. The cell suspension was then spun down and cell pellets were lysed for 30 min in lysis buffer [50 mM Tris (pH 7.5), 0.5 M NaCl, 1.0 mM EDTA (pH 7.5), 10% glycerol, 1 mM BME, 1% IGEPAL-630 and proteinase inhibitor cocktail (Roche)] and centrifuged at 12 000 g for 10 min. The supernatants were removed and placed in new Eppendorf tubes for western blot analysis. Proteins from the RSC96 cells were separated in 12% gradient SDS-PAGE and transferred onto nitrocellulose membranes. Non-specific protein binding was blocked in blocking buffer at RT for 1 h (5% milk, 20 mM Tris-HCl, pH 7.6150 mM NaCl and 0.1% Tween 20). The membranes were blotted with specific antibodies and incubated in 4°C blocking buffer overnight. For repeated blotting, nitrocellulose membranes were stripped with Restore Western blot stripping buffer (Pierce Biotechnology, Inc, Rockford, IL, USA) at room temperature for 30 min. Densitometric analysis of immunoblots was performed using the AlphaImager 2200 digital imaging system (Digital Imaging System, CA, USA). Experiments were performed in triplicate.

### 2.8. Zymography

MMP-2 and MMP-9 activity was determined using gelatin zymography. RSC96 cells were treated with earthworm extract at different times. After incubation for 0, 4, 8, 12, 16, 20 and 24 h, the cell medium was collected. Sample medium was electrophoresed on an 8% polyacrylamide gel containing 0.1% gelatin. After electrophoresis, the gel was washed for 30 min two times in washing buffer (2.5% Triton X-100). The gel was then incubated in incubation buffer (1% NaN_3_; 2M Tris-HCl, pH 8.0; 1 M CaCl_2_) at 37°C for 24 h with shaking and subsequently stained with Coomassie blue. The presence of MMP-2 and MMP-9 gelatinolytic activity was identified as clear bands on a blue background after destaining.

### 2.9. siRNA

Double-stranded siRNA sequences targeting MEK and p38 mRNAs were obtained from Dharmacon. A non-specific duplex (Dharmacon) was used as a control. RSC96 cells were cultured in 100-mm well plates in DMEM without FBS and transfected with double-stranded siRNA using the DharmaFECT Duo Transfection Reagent (Dharmacon) according to the manufacturer's instructions. To assess gene silencing, the ERK1/2 ans p38 protein level was detected by western blot.

### 2.10. Statistical Analysis

Statistical differences were assessed using one-way ANOVA. *P* < .05 was considered statistically significant. Data were expressed as the mean  ±  SEM.

## 3. Results

### 3.1. Effects of Earthworm on Cell Viability

**Figure 1:**
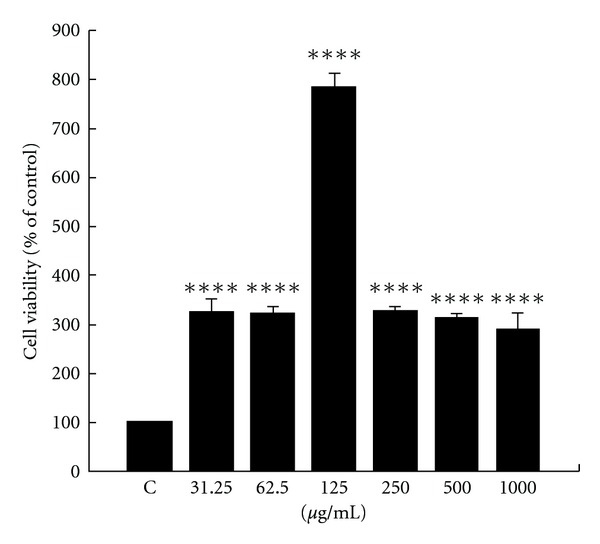
Effect of earthworm extract on RSC96 cell viability. Schwann cells were treated with 0–1000 *μ*g/ml earthworm extract for 24 h. Cell viability, measured by MTT assay, was described under materials and methods section. Data are shown as the mean of three independent experiments  ±  SE. **** denote significant differences from control values with *P* < .0001.

We evaluated the proliferative effect of earthworm extract on the regenerative ability of RSC96 cells. During experiments, we first observed the effect of various earthworm extract concentrations (0, 31.25, 62.5, 125, 250, 500 and 1000 *μ*g/ml) on cell viability for 24 h. We found that cellular viability was significantly elevated with the concentration at 125 *μ*g/ml for 24 h (Figure 1). However, some of the effects were reversed back to the basal level at doses of 250–1000 *μ*g/ml, indicating that the toxicity effect of earthworm extract could occur at high concentrations. These results may elucidate that treatment with 125 *μ*g/ml earthworm extract appears to induce cell proliferation. Moreover, the viability of ethanol-treated Schwann cells was not affected (data not shown). 

### 3.2. Earthworm Promotes the Migration of RSC Cell

**Figure 2:**
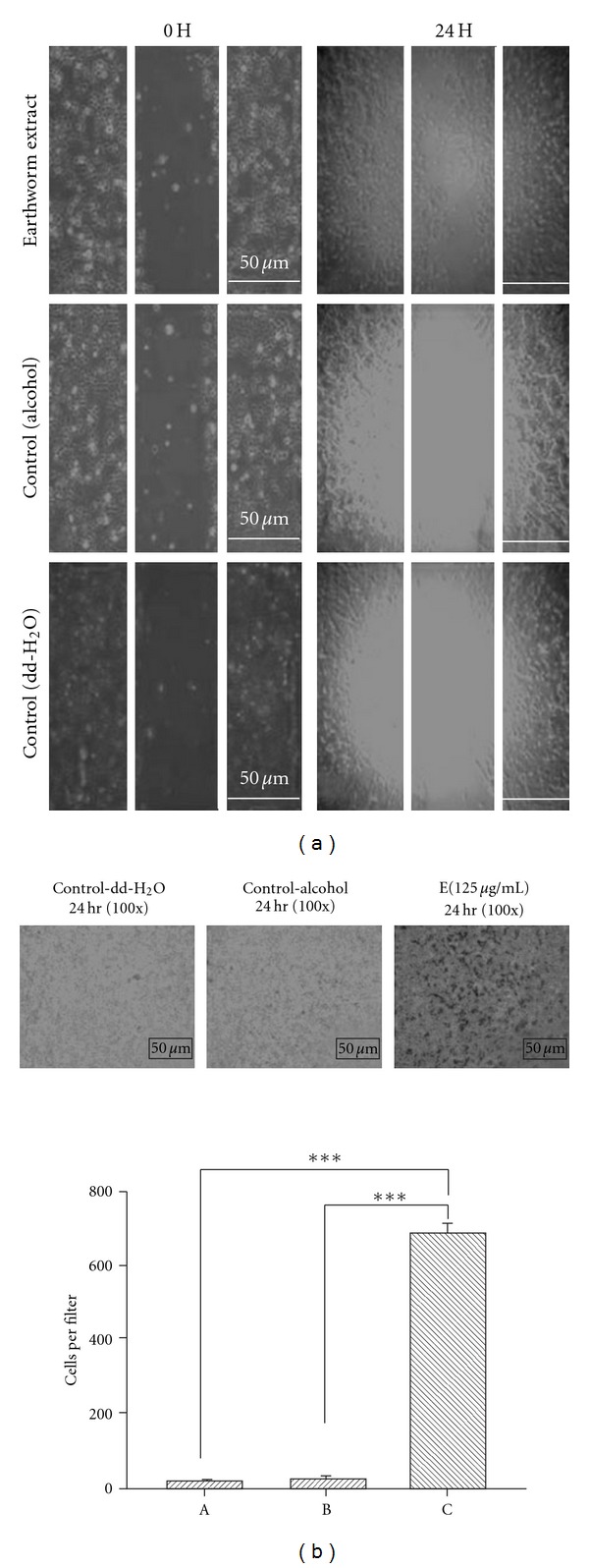
The migrative effect of earthworm extract on RSC96 cell. Schwann cells were treated with 125 *μ*g/ml earthworm extract for 24 h as indicated, and cell migration as measured by wound healing analysis (a) and Boyden chambers (b) were described under materials and methods. Data are shown as the mean of three independent experiments  ±  SE. *** denote significant differences from control values with *P* < .001.

The property of RSC96 cells to migrate along the growth direction, leading to regenerating damaged nerves is important in helping the damaged peripheral nerve regeneration [38]. Therefore, we further performed an *in vitro* wound-healing experiment to evaluate the migration potential of RSC96 cells. As shown in Figure 2(a), treatment with 125 *μ*g/ml earthworm extract for 24 h significantly enhanced the mobility of RSC96 cells. Moreover, the same treatment also strongly promoted cell migration in either water- or ethanol-treated controls in Boyden chambers. These samples were stained and counted (Figure 2(b)). These observations provide evidence of earthworm extract-induced RSC96 cell proliferation and migration to enhance nerve regeneration. 

### 3.3. Role of MAPKs in Earthworm Induced RSC Cell Migration

**Figure 3:**
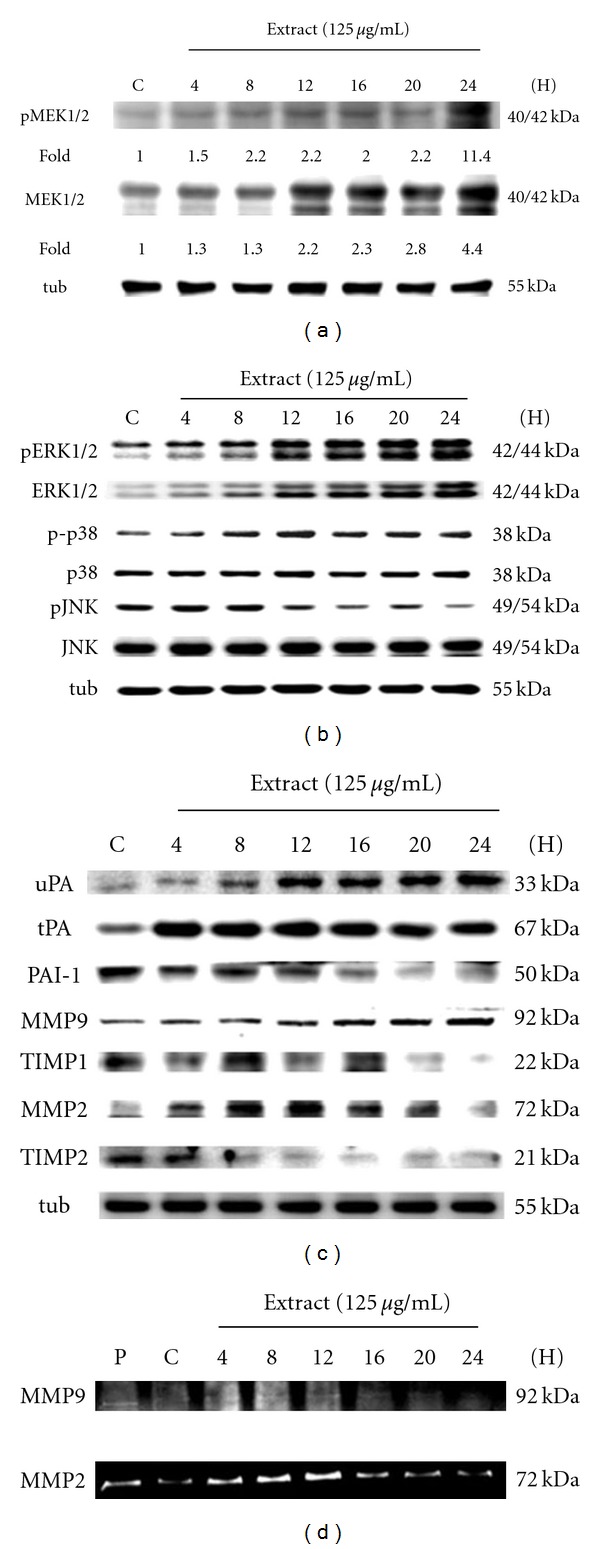
MAPK signal pathway activation time course for Schwann cells treated with earthworm extract. Schwann cells were treated with 125 *μ*g/ml earthworm for different time as indicated, and then the cells were harvested and extracted for the western blot analysis. The protein expression of the MAPK signal pathway was determined by western blot (a)–(c). *α*-Tubulin was used as a loading control. Further to confirm the MMP-9 and MMP2 activity by gelatin zymography (d). Lane 1: FBS as positive marker (P).

We further examined the Schwann cell migration mechanisms using earthworm extract. As shown in Figure 3(c), the protein levels were measured using western blotting. The MAPK signal pathway activation mechanism results for RSC96 cells treated with earthworm extract show a time course effect. In contrast to ERK1/2 and p38, JNK phosphorylation began to decline in the same time period as ERK and p38 activity increased. We suggest that earthworm extract could induce the phosphorylation of ERK1/2 and the p38 signal pathway to promote migration in Schwann cells. Moreover, uPA and tPA proteins also rapidly increase and conversely the PAI-1 protein level gradually decreases. Interestingly, the maximal tPA expression was observed early at 4 h in Schwann cells treated with earthworm extract. RSC96 cells exposed to earthworm extract also induced the expression of MMP9 and MMP2 proteins, their activity (Figure 3(d)), and decreased TIMP1 and TIMP2 levels. These results indicate that earthworm extract might be mediated through the activation of ERK1/2 and p38 pathways to induce PAs and MMP2/9, resulting in Schwann cell migration. 

### 3.4. RSC Cell Migration Enhanced by Earthworm Is ERK1/2 and p38 Signalingdependent *In Vitro*

**Figure 4:**
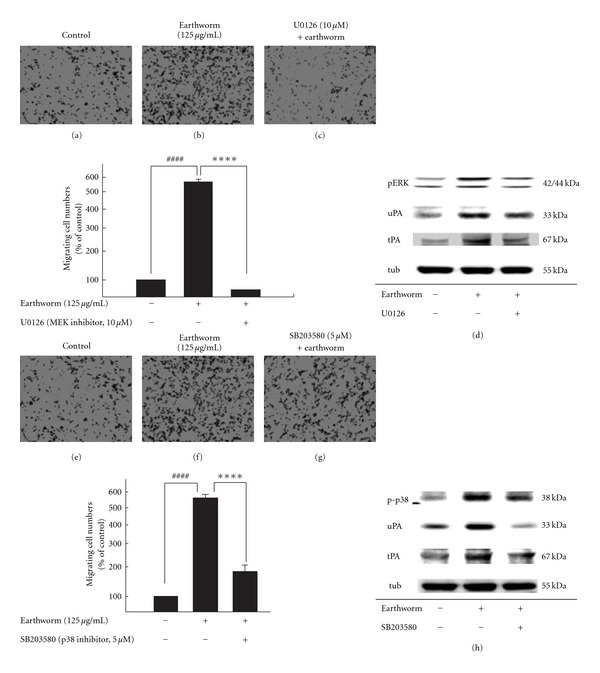
Earthworm extract effects on Schwann cell migration was ERK1/2 and p38 signaling dependent. RSC cells were pretreated with U0126 or SB203580 for 1 h, then treated with 125 *μ*g/ml earthworm extract for 24 h. After incubation with earthworm extract, migration was assayed using Boyden chambers ((a)–(c), (e)–(g)). Cells that migrated through the membrane were counted using a counting grid fitted into a phase contrast microscope eyepiece. The results shown are the mean number of migrating cells per field performed in triplicate. Western blot analysis of many protein levels, as indicated, in RSC cells treated with earthworm extract. ((d) and (h)). ^####^ denote significant differences from control values with *P* < .0001. **** denote significant differences treated with earthworm extract only values with *P* < .0001.

**Figure 5:**
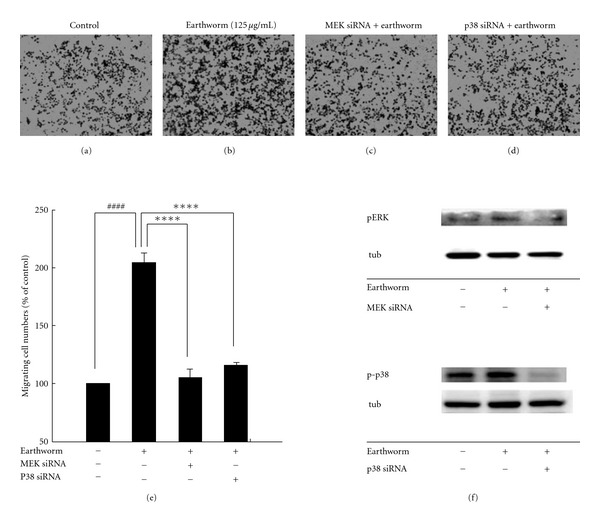
ERK1/2 and p38 signaling required for earthworm-induced cell migration. RSC cells were transiently transfected with ERK1/2 siRNA (100 nM) or p38 MAPK siRNA (100 nM) for 8 h, then treated with 125 *μ*g/ml earthworm extract for 24 h. After incubation with earthworm extract, migration was assayed using Boyden chambers ((a)–(d)). Cells that migrated through the membrane were counted using a counting grid fitted into a phase contrast microscope eyepiece. The results shown are the mean number of migrating cells per field performed in triplicate (e). After treatment with earthworm extract for 24 h to prepare protein lysates and following western blot assay using anti-pERK or p-p38 antibody (f). ^####^ denote significant differences from untreated control values with *P* < .0001. **** denote significant differences from treated with earthworm extract only values with *P* < .0001.

It has been demonstrated that earthworm extract can significantly activate ERK1/2 and p38. We then examined whether earthworm-induced cell migration indeed occurred through ERK1/2 and p38 MAPK. We used specific MAPK cascade inhibitors: U0126, and SB203580. Schwann cells were pretreated with U0126 and SB203580 pharmacological inhibitors, followed by incubation with earthworm extract at 125 *μ*g/ml concentration for 24 h. Our data reveal that ERK1/2 and p38 inhibition activity significantly blocked earthworm-increased cell migration (Figures 4(a)–4(c) and Figures 4(e)–4(g)). The downstream substrate of ERK1/2 and p38 in the signaling activated by earthworm extract was blocked (Figures 4(d) and 4(h)). To further confirm the role of ERK and p38 in earthworm extract enhanced cell migration, we chose to deplete ERK and p38 using siRNA (Figure 5). Most importantly, reduced ERK expression with siRNA also markedly attenuated earthworm-induced migration compared with the control. Similarly, p38 siRNA at the same concentration did reduce cell migration. Western blots showed that there was a significant reduction in the pERK and p-p38 protein level in Schwann cells transfected with MEK or p38 siRNA. Consistent with the inhibitor effect, cell migration induced by earthworm extract was clearly reduced in the presence of ERK1/2 and p38 MAPK siRNA. Therefore, our results demonstrate that earthworm extract-induced Schwann cell migration occurs by activating the PAs and MMPs dependent on the ERK1/2 and p38 MAPK pathways. 

## 4. Discussion

**Figure 6:**
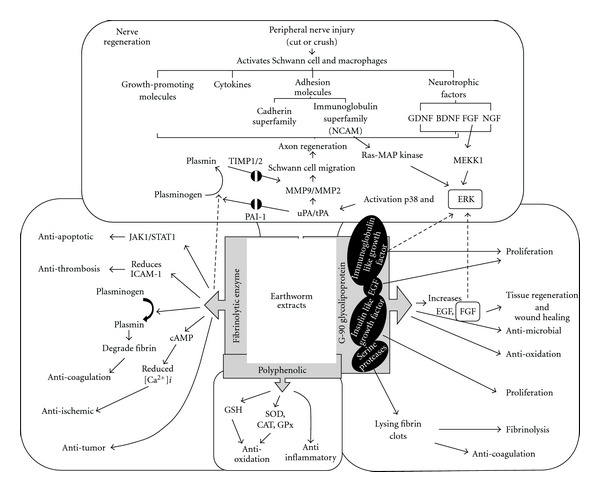
Schematic model of earthworm extract migrative effects on RSC96 Schwann cells. Stimulation of Schwann cells with earthworm activates ERK1/2 and p38 signaling, leading to upregulating uPA and tPA, and contributing to activating MMP9 and MMP2, enhancing the Schwann RSC96 cell migration. NCAM, neural cell adhesion molecule; GDNF, glial cell line-derived neurotrophic factor; BDNF, brain-derived neurotrophic factor; FGF, fibroblast growth factor; NGF, nerve growth factor; ICAM-1, intercellular adhesion molecule-1; JAK1/STAT1, janus kinase1/signal transducers and activators of transcription1; GSH, glutathione; GPx, glutathione peroxidase; CAT, catalase; EGF, epidermal growth factor. *Dotted lines* indicate the hypothetical molecular mechanism of the bioactive compound present in earthworm powder.

The overall aim of this study was to investigate the mechanism in which earthworm extract regulates Schwann cell migration. We demonstrated a specific signaling migration pathway in earthworm-stimulated Schwann cells, inducing the activation of uPA and tPA mediated through the ERK1/2 and p38 (Figure 6). When cells-treated earthworm extract resulted in ERK1/2 and p38 phosphorylation, the expression of uPA and tPA occurred in a time-dependent manner, leading to elevated MMP9 and MMP2 levels and activity. Using inhibitors and siRNA, the migrative effects of earthworm extract on Schwann cells were further identified to beERK1/2 and p38 signaling dependent. 

Chinese herbal medicines have attracted a great deal of attention as alternative and supplemental medicines [39]. For thousands of years the earthworm has been used as a drug for various diseases in China and the Far East [34]. Schwann cells in the injured nerve area migrate and form a Büngner band, supporting axonal regrowth [2]. The function of earthworm extract on nerve regeneration is unknown and migratory Schwann cell mechanism with earthworm extract treatment is totally obscure. Recently studies have demonstrated that MAPKs, including JNK, p38 and ERK1/2, play crucial roles in nerve cell migration [40]. This further demonstrated that earthworm extract stimulated ERK1/2 and p38, but not JNK activation in a time-dependent manner, leading to Schwann cell migration. Earthworm-induced Schwann cell motility and phosphorylation of ERK1/2 and p38 were both attenuated by pretreatment with MEK1/2 (U0126) and p38 (SB203580) inhibitors. Transfection with siRNA of MEK1/2 and p38 significantly reduced migration in response to earthworm extract in Schwann cells as well. These assays, allow us to examine the individual steps in the complex signaling cascades and clearly illustrate direct earthworm extract effects on Schwann cell migration.

It has been reported that the highly expressed uPA in the epidermis of damaged tissue is regulated by the fibroblast growth factor (FGF-2) which affects MAPK kinase (MEKK-1) and MEKK-1's downstream ERK1/2 for controlling uPA expression [41]. The p38 MAPK pathway also participates in endothelial cell migration by regulating uPA expression [42].

We further identified that earthworm extract enhances uPA expression directly through the ERK1/2 and p38 signaling pathway. To promote migration, cells secrete proteases that are thought to degrade matrix molecules and cell adhesion. These proteases include tPA and Upa [14]. In contrast to PAs, PAI-1 is thought to be the major inhibitor. Our data clearly shows that the phosphorylation of ERK1/2 and p38 accompanies the increased expression of uPA. Conversely, PAI-1 expression is gradually decreased. Interestingly, tPA levels reached the maximal early at 2 h and then started to slightly decline until 20 h. We suggest that the maximum expression of tPA occurred early following 4-h treatment, because tPA is the main PAs in the nerve growth cones [43]. tPA is consequently rapidly induced by earthworm extract. Pittman and Dibenedetto reported that overexpressing tPA regenerates neurites to a greater extent and migrates faster than control cells in complex extracellular matrix [44]. Ulfhammer et al. found that tPA activation could be mediated through p38 pathways, leading to an increase in tPA expression [45]. Our experiments further show that SB203580 inhibited p38 phosphorylation and suppressed tPA protein expression in Schwann cells. Thus, tPA activation occurs not only through ERK1/2 activation, but also through the p38 signaling pathway.

The development and regeneration of the PNS is highly dependent on the migration of Schwann cells and the extension of axons toward their distant targets. PAs are associated with several neural cell types where they are believed to mediate localized degradation of the extracellular matrix (ECM), thus facilitating cell motility [46]. Degradation of the ECM is associated with the development of tumor metastasis and neuron tissue growth. One of the key regulators of this process is the serine protease, uPA, acting on a wide variety of ECM components [47]. The cell proliferation [48] and angiogenesis [49] processes are events involving uPA catalytic ECM degradation. Interestingly, several studies have reported that Schwann cells also produce some growth factors, which are crucial for peripheral nerve repair, such as FGF-2. FGF-2 is one of the mediators of uPA activity, and induces signals to control uPA expression and function [41]. We suggest that ERK1/2 and p38 phosphorylation could promote uPA expression in earthworm extract-treated cells, which could be mediated through the modulation of FGF-2. Another family of proteases, the matrix metalloproteases (MMPs), are also implicated in peripheral nerve regeneration [50], and involved in many cell migration phenomena and produced by many cell types, including neurons [51]. MMPs are secreted as inactive molecules and require activation via other proteases [52]. Plasmin, activated by tPA or uPA, can activate MMP-9 and MMP-2 [15]. Our results also show the elevated protein expression and activity of MMP2 and MMP9.

Earthworm extract is commonly used in Chinese medicine. It has a dense nutritional content because of its soil-based origin [23]. Lumbrokinase (LK) is a group of six novel proteolytic enzymes derived from the earthworm *Lumbricus rubellas* [53], as a strong fibrinolytic enzyme [54]. Recent studies have shown that the fibrinolytic enzymes could dissolve blood fibrin clots and inhibit platelet activation and aggregation [55]. Its therapeutic and preventive effects for thrombosis-related disease have been clinically confirmed [56]. A novel fibrinolytic enzyme also found from *Pheretima aspergillum* can dissolve human thrombi and fibrin directly and strongly, and also activate human plasminogen to plasmin. This enzyme showed little toxic side effects in animal tests [33]. Several experiments have indicated that earthworm fibrinolytic enzyme acts as a plasminogen activator [57], suggesting a tPA-like function [58]. A biologically active glycolipoprotein extract from a whole earthworm tissue homogenate was isolated and named G-90 [59]. It is neither mutagenic nor cancerogenic [60]. It was recently shown that G-90 is neither allergic nor toxic, and possesses antibacterial activity [61] and antioxidative effects [62]. G-90 exhibits many biological functions important in the proliferation [63] and adhesion [64] of cells. The G-90 mixture contains insulin like growth factors, immunoglobulin-like growth factor, serine proteases and epidermal growth factor (EGF) [6, 35, 59, 61, 63, 64]. Furthermore, it also contains molecules to activate signal transduction pathways, which *in vivo* help the tissue regeneration process; that is, wound healing. *In vivo* experiments showed stimulation of EGF and FGF synthesis in skin wounds using G-90 [65]. In cell cultures, after treatment with H_2_O_2_ for 4 h, G-90 allows the cells to recover and stimulated their growth. G-90 could be a useful wound-healing agent [62]. The neural cell adhesion molecule (NCAM) is a member of the immunoglobulin superfamily. Several studies showed that NCAM-induced neurite outgrowth depends on Ras-mitogen-activated protein (MAP) kinase pathway activation [66]. NCAM-dependent cell migration to fibronectin required an intact MEK-ERK signaling pathway [67]. The adhesins of the immunoglobulin superfamily from earthworm extract could promote migration by MEK-ERK pathway activation. These bioactive compounds may indirectly cause ERK activation or directly activate plasminogen to plasmin by fibrinolytic enzyme, resulting in Schwann cell migration promotion in nerve regeneration. Based on these facts, we suggest that earthworm extract or some of its components could have cell migration promotion potential. Our results demonstrate that earthworm extract can stimulate Schwann cell migration and up regulate PAs and MMP2/9 expression mediated through the MAPK pathways, ERK1/2 and p38. Further analyses are needed to determine the presence of bioactive compounds that promote cell migration in earthworm extract.

The findings of our study provide another neuron regeneration novel function. However, the nerve growth-suppressing action by high doses of earthworm extract at concentrations of 250–1000 mg/ml, indicates that an excessive earthworm extract load in the medium could provoke an adverse response to neuron regeneration recovery. The data agree with the results of Boyd and Gra's study [68], demonstrating that excessive supplement could saturate the neurotrophin receptor, p75, to block the neuron regrowth promoting function. Excessive nerve growth factor administration could delay the neurotrophic factor, growth-associated protein 43 (GAP 43) induction and early phase peripheral nerve regrowth [69]. Therefore, an appropriate dose of earthworm extract should be carefully selected to reach the highest potential for enhanced Schwann cell migration. Earthworm extract might serve as a promising migration inducing and/or therapeutic drug for nerve regeneration.

## Funding

China Medical University (CMU95-058, CMU95-060, CMU96-102 and CMU97-CMC-007).
